# Older Adults’ Participation in Social Activities Differ by Whether They Rent or Own Their Homes

**DOI:** 10.1093/geroni/igaf122.3006

**Published:** 2025-12-31

**Authors:** Ebenezer Kyeremeh, Justine Sefcik, Rose Ann DiMaria-Ghalili, Chanee Fabius, Safiyyah Okoye

**Affiliations:** Drexel University, Philadelphia, Pennsylvania, United States; Drexel University College of Nursing and Health Professions, Philadelphia, Pennsylvania, United States; Drexel University, Drexel University, Pennsylvania, United States; Johns Hopkins University, Baltimore, Maryland, United States; Drexel University, Philadelphia, Pennsylvania, United States

## Abstract

Prior research has substantiated social engagement as a beneficial determinant of psychological and social well-being in later life. International studies find that renting in congregate housing can provide opportunities for social participation among older adults; however, there has been little study of whether social participation varies between older adults who rent vs own their homes in the U.S. Understanding the relationship between housing tenure (i.e., renting vs owning) and social participation can inform programs and policies, tailored to housing tenure status, to support social engagement. We employed a cross-sectional design using nationally representative data from N = 5137 community-dwelling older adults (≥65 years) from the 2022 National Health and Aging Trends Study (NHATS) to explore the association between owning or renting and social participation (measured as self-report of ≥ 1 of the following in the previous month: visiting family/friends, attending religious services, attending club meetings/group activities, going out for enjoyment, or doing volunteer work). We found a higher proportion of homeowner participants reporting social participation (96.7%) compared to renters (89.2%). In an unadjusted logistic regression model, renters had much lower odds of participating in social activities than owners (OR 0.28, 95% CI: 0.20, 0.40). The association weakened after adjusting for sociodemographic (age, gender, education, race, financial strain, income, living arrangement) and health characteristics (chronic conditions and cognitive, functional, and sensory impairments) (OR for renters 0.71, 95% CI: 0.50, 1.02). Further studies are needed to describe barriers to social participation among older adult renters, focusing on sociodemographic, financial, and health factors.

